# Networking the *Leishmania* research community for the development of novel anti-leishmanial intervention strategies

**DOI:** 10.1186/1753-6561-5-S1-L8

**Published:** 2011-01-10

**Authors:** Gerald F Späth

**Affiliations:** 1 G5 Virulence Parasitaire, Institut Pasteur, CNRS URA 2581, INSERM U1010, 75015 Paris, France

## 

The EU-sponsored LEISHDRUG consortium uses a highly interdisciplinary approach to reveal *Leishmania* signaling molecules associated with virulence of the pathogenic amastigote stage. LEISHDRUG coordinates the efforts of 13 partners across eight nations, including four institutes of the Institut Pasteur international network, with the major aim to exploit the *Leishmania* kinome for anti-parasitic drug development.

The consortium is based on three clusters with each two interactive scientific work packages that together follow the major stages of the drug development process, including identification of hit compounds and target kinases, hit-to-lead validation and lead characterization. We use innovative drug screening concepts not applied previously on parasitic systems, including visual high-content screening to discover compounds capable to kill intracellular *Leishmania* amastigotes without deteriorating the host cell. This phenotype-based strategy relies on fluorescent parasites and macrophages as read-outs and will allow simultaneous assessment of anti-leishmanial activity and host cell toxicity under physiological conditions. We apply a target-based strategy utilizing recombinant *Leishmania* protein kinases for inhibitor identification and structure-guided drug design. The identification of appropriate target kinases, with only limited homology to their mammalian counterparts relies on in silico analysis by applying novel bioinformatic tools developed by consortium members, and in vitro assays based on their phospho-transferase activity towards recombinant *Leishmania* phospho-proteins.

The major objectives of our consortium are (i) to screen small molecule and peptide libraries for hit compounds with leishmanicidal activity using phenotype- and target-based strategies, (ii) to identify anti-parasitic lead compounds and assess their pharmacokinetic profiles using cell-culture and experimental infection models for leishmaniasis, and (iii) to initiate lead optimization by structure-based drug design.

